# Microelectrode arrays, electrosynthesis, and the optimization of signaling on an inert, stable surface

**DOI:** 10.3762/bjoc.18.156

**Published:** 2022-10-20

**Authors:** Kendra Drayton-White, Siyue Liu, Yu-Chia Chang, Sakashi Uppal, Kevin D Moeller

**Affiliations:** 1 Washington University in Saint Louis, Saint Louis, Missouri 63130, United States

**Keywords:** calibration of binding curves, electrochemical signaling, microelectrode array

## Abstract

Microelectrode arrays are powerful tools for monitoring binding interactions between small molecules and biological targets. In most cases, molecules to be studied using such devices are attached directly to the electrodes in the array. Strategies are in place for calibrating signaling studies utilizing the modified electrodes so that they can be quantified relative to a positive control. In this way, the relative binding constants for multiple ligands for a receptor can potentially be determined in the same experiment. However, there are applications of microelectrode arrays that require stable, tunable, and chemically inert surfaces on the electrodes. The use of those surfaces dictate the use of indirect detection methods that are dependent on the nature of the stable surface used and the amount of the binding partner that is placed on the surface. If one wants to do a quantitative study of binding events that involve molecules on such a stable surface, then once again a method for calibrating the signal from a positive control is needed. Fortunately, the electrodes in an array are excellent handles for conducting synthetic reactions on the surface of an array, and those reactions can be used to tune the surface above the electrodes and calibrate the signal from a positive control. Here, we describe how available Cu-based electrosynthetic reactions can be used to calibrate electrochemical signals on a polymer-coated electrode array and delineate the factors to be considered when choosing a polymer surface for such a study.

## Introduction

Microelectrode arrays composed of collections of electrodes that can each be individually addressed are powerful platforms for monitoring interactions between a protein target and small molecules [1–8]. In these efforts, molecules are either placed or synthesized by specific, selected electrodes in the array, and then the associated electrodes are used to monitor any subsequent binding events involving those molecules [8–9]. The method frequently relies on the chemistry used to place or synthesize the molecules by the surface of the electrodes in the array because it is the nature of those molecules that defines the biological interactions that can be studied [10–11]. As discussed below, such efforts need to not only control the selectivity of the reactions for specific, predetermined sites on the array, but also control the surface concentration of the molecules placed at those sites.

In the majority of such studies, the molecules to be added to the electrodes in an array are attached directly to the array either through the use of a self-assembled monolayer or direct functionalization of a carbon electrode [12–13]. Following the synthetic chemistry used to place molecules on a microelectrode array, binding events on the surface of the array are typically monitored by using the electrodes in the array to measure current changes that are caused by that event. The method is ideal in that it allows for the "real-time" detection of the binding event. In many cases, these measurements are made using an electroactive group that is incorporated into, or found naturally in, either the molecule placed on the surface of the electrodes or in the protein target. The binding event changes the distance between this electroactive group and the associated electrodes resulting in a change in the ability of the electrode to either oxidize or reduce the group. This causes a change in the current measured for the redox reaction [14]. Alternatively, the binding event can be detected with an indirect, impedance-based method. This method works by inserting a functionalized electrode into a solution containing a redox mediator. The mediator is oxidized at the electrodes in the array and reduced at a remote counter electrode. This results in a current that can be measured at each electrode in the array. The protein target is then added to the solution above the array, and when it binds one of the molecules fixed to the surface of a set of electrodes in the array it causes a change in the current to be measured at those electrodes. This change in current can be recorded and the binding event monitored. By varying the concentration of the protein in solution and recording the corresponding change in current, a binding curve for the interaction can be generated.

**Figure 1:**
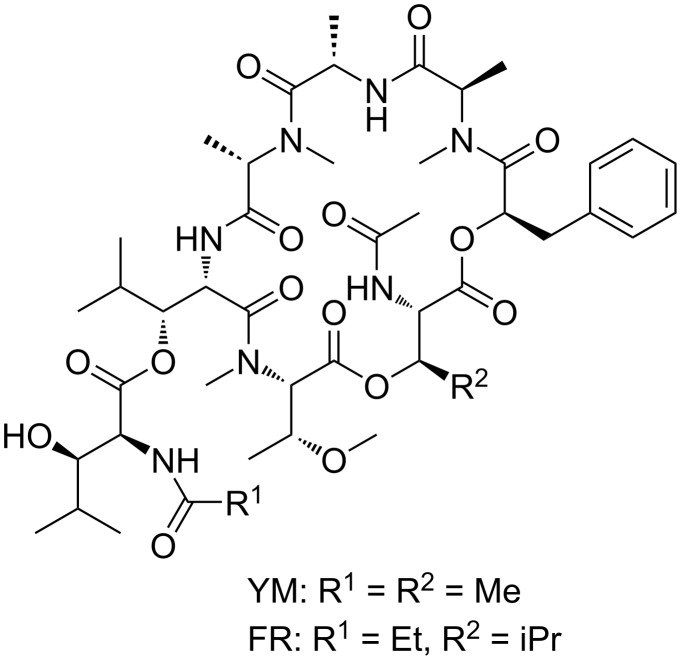
Natural products YM-254890 and FR-900359.

While this approach can be very effective, it has limitations. We are interested in using microelectrode arrays to guide synthetic efforts to build molecular probes for protein active sites. For example, consider a pair of molecules (Figure 1) that selectively inhibit the Gq_11_-signaling pathway in cells [15–16]. The molecules are complex cyclic peptides, and the construction of analogs of the molecules to probe the basis of their activity is a significant challenge. In an ideal situation, the design and synthesis of those analogs would be informed by biological data that was gathered iteratively with the synthetic efforts. That scenario requires each new analog synthesized to be rapidly and accurately evaluated for activity. Microelectrode arrays that can be used to quickly gather real-time data on binding events appear ideal for such an iterative study. However, to use a microelectrode array in this fashion requires that the molecules being synthesized be added to the surface of an array as they become available, and then the growing library analyzed in a manner that allows the relative binding of the new analog to be compared to previous analogs and the natural products.

**Figure 2:**
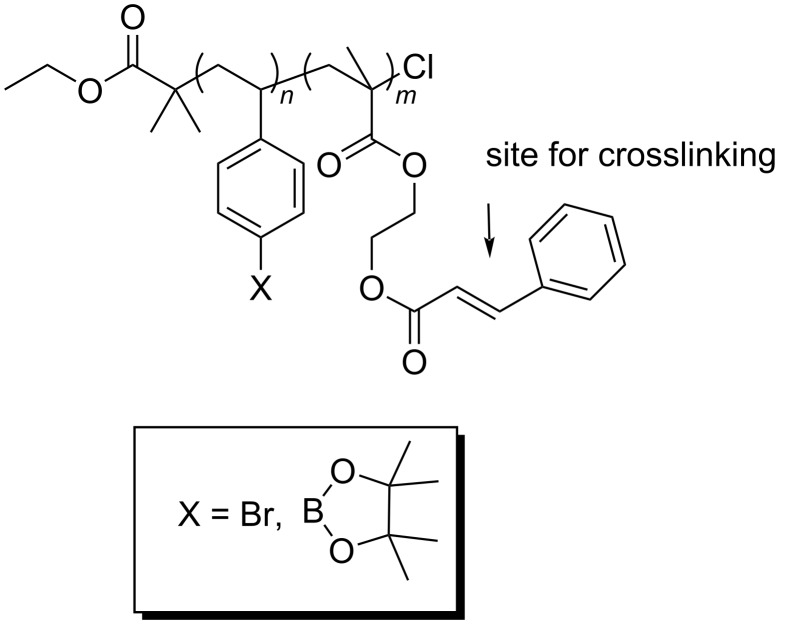
Diblock copolymers used for coating microelectrode arrays.

For the strategy to be viable, the surface coating on the electrodes must be very stable over time, chemically inert to the reactions used to place molecules on the array, and tunable so that nonspecific interactions can be minimized for a variety of biological targets. Self-assembled monolayers do not have this stability, and strategies that chemically modify carbon electrodes do not offer the chemical flexibility or surface tunability needed. Hence, an alternative strategy is required. This led to the development of polymer-coated arrays that capitalize on the versatility of diblock copolymers as surface coatings (Figure 2) [17–19]. In the polymer, one of the blocks is used to place attachment sites for molecules above the electrodes (the arylbromide or arylborate block in the polymer shown), and the other block is used to provide a site for cross-linking the polymer in order to render the surface more stable once it is coated onto an array (the cinnamate block is used to make a polymer network through photochemical cross-linking). The use of the borate ester surface provides a tunable surface that can be used to help prevent non-specific binding and minimize biofouling possibilities [18]. While the use of a diblock copolymer coating offers the desired stability advantage, it also rules out the use of both direct methods for conducting synthetic transformations on the array and the direct detection methods in the subsequent binding studies. The polymer network is not permeable to molecules tethered to its surface or proteins in solution.

**Figure 3:**
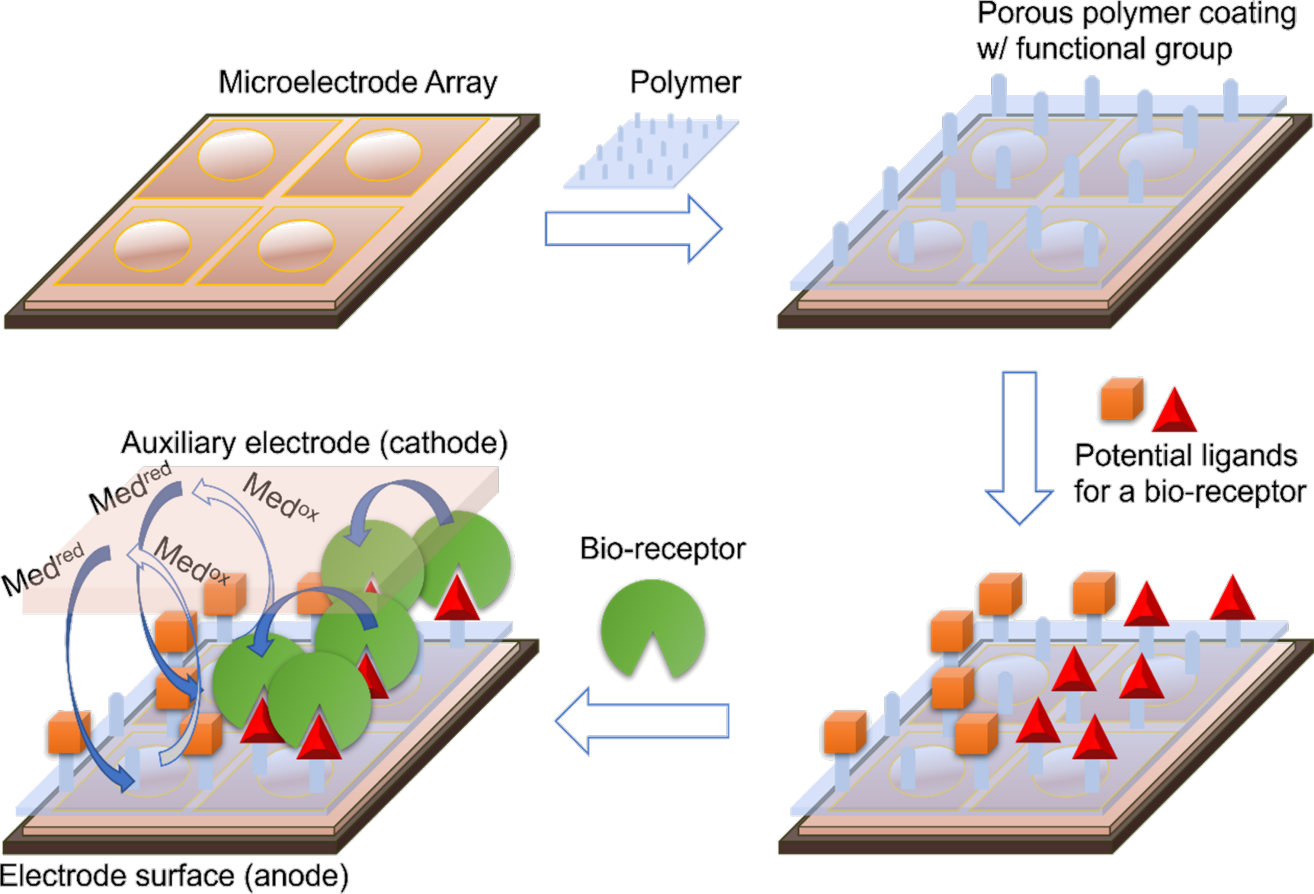
An indirect method for detecting binding events.

The synthetic problem was solved by using the electrodes in an array to generate chemical reagents and catalysts and then confining those reagents and catalysts (and the reactions they mediate) to the surface of the electrodes for their generation [10–11]. For the subsequent signaling experiments, the impedance-based approach outlined above has proven effective with polymer-coated electrodes (Figure 3). As described, this method works by inserting a functionalized electrode into a solution containing a redox mediator and then monitoring the current associated with a redox mediator. The method is different relevant to the traditional methods lacking the polymer surface in that the current is dependent on changes to the polymer coating; a situation that can lead to analogous drops in current to methods involving molecules bound directly to an electrode surface or increases in current if a binding event helps to swell a polymer.

**Scheme 1:**
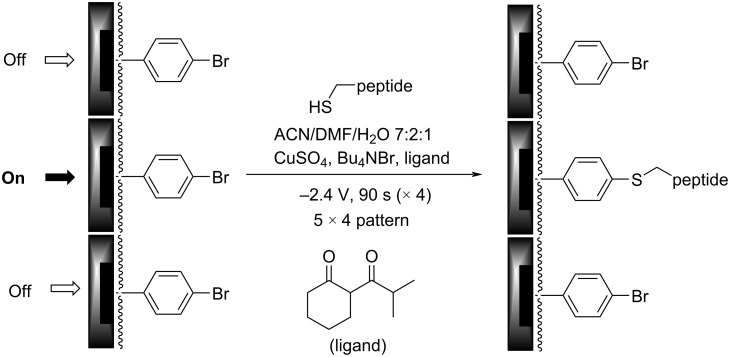
A Cu(I)-catalyzed cross-coupling reaction on an array.

**Figure 4:**
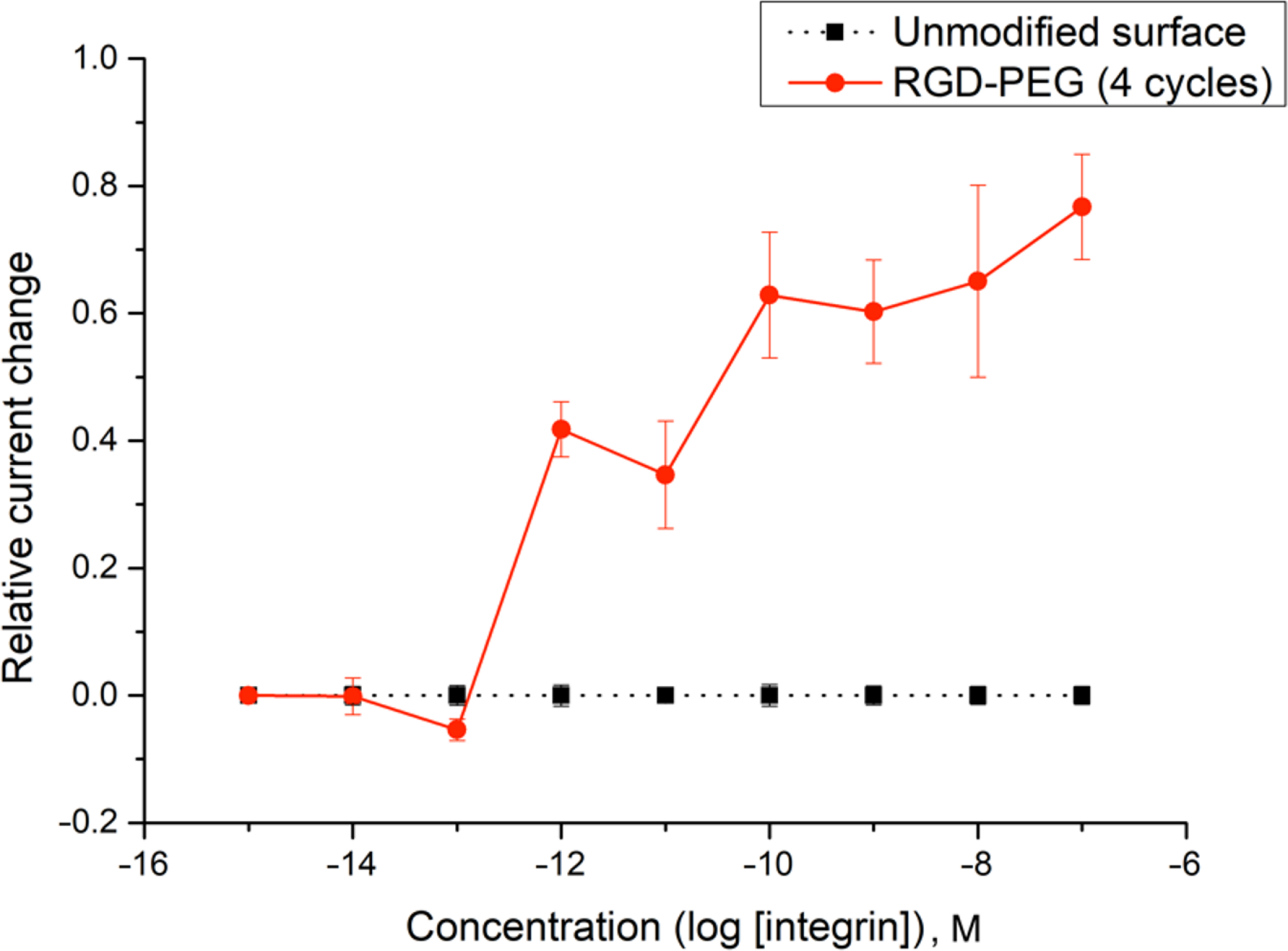
A model study using an RGD peptide (C-PEG6-GGRGDGP) and integrin receptor (α5,β1).

The utility of this approach for a polymer-coated electrode was verified by looking at several small molecule–protein interactions [5–7]. For example, a binding curve generated for the interaction between an RGD-peptide (C-PEG6-GGRGDGP) and its integrin (α5,β1) target is shown in Figure 4 below. A PEG-linker was added to the RGD-peptide so that the peptide would not be buried in the polymer coating the surface of the array, a scenario that would make it unavailable for binding to the integrin receptor in the subsequent analytical experiment. The PEG-6-linker was selected because it was effective and readily available from commercial sources. This linker was functionalized with a cysteine on the end opposite the peptide so that the thiol group in the sidechain could be used to place the molecule the array with the use of an electrochemically initiated Cu(I)-catalyzed cross-coupling reaction (Scheme 1) [9]. To this end, the Cu(I) catalyst needed for the reaction was generated at the electrodes by the reduction of Cu(II). Confinement of the Cu(I) catalyst to the selected electrodes was accomplished with the use of oxygen in solution. The oxygen oxidized any Cu(I) before it could migrate to a non-selected electrode. The electrodes selected for the reduction were cycled on an off with each cycle turning the electrode on for 90 s and then off for 180 s. This was done to tune the rate of Cu(I) generation to match the rate of Cu(I) oxidation in solution and in so doing optimize confinement of the Cu(I) catalyst to the selected electrodes. Longer "on-times" would lead to more reagent generation making it harder for the confinement strategy to keep up. The result is a loss in confinement. It is important to point out that the reaction shown in Scheme 1 is not a typical electrosynthetic reaction. The cross-coupling reaction shown is a Cu(I)-catalyzed transformation that requires no recycling of a reagent; there is no net oxidation or reduction involved. Instead, on the array the catalyst is purposely destroyed in the solution above the electrodes and then regenerated electrochemically so that the location of the reaction can be confined to only desired sites on the array. Unlike the synthesis of a complex molecule, the synthesis of a complex, two-dimensional addressable surface requires a new type of selectivity – "site-selectivity". The use of electrosynthesis is essential for obtaining this selectivity.

With the substrate on the surface of the array, a hydroquinone/quinone redox couple was then used for the subsequent signaling experiment [20]. The hydroquinone/quinone redox couple has superior stability to the iron-based systems used previously [20], and its use leads to more reproducible binding curves. To generate the binding curve shown in Figure 4, the concentration of the integrin receptor was varied (and plotted on the *x*-axis) and then the current measured for the redox couple by CV (current = peak current for the oxidation − peak current for the reduction) for each concentration of the receptor. Blocks of 12 electrodes were used for recording the current with each data point in Figure 4 representing the average current measured at three such blocks with the error bars indicating the range in the data. The use of the method to probe interactions between a v107-peptide and its targeted VEGF receptors proved equally effective [7].

The binding curve shown in Figure 4 did indicate a surface interaction that was stronger than the solution-phase nanomolar *K*_d_-value known for the RGD–integrin binding event. This was not viewed as an issue since we could see the whole binding curve, and the arrays are used to examine relative binding events between molecules on the array and not determine absolute binding constants in the absence of a positive control. In this case, the positive control needed for any subsequent study was cleary visible and quantifiable.

**Figure 5:**
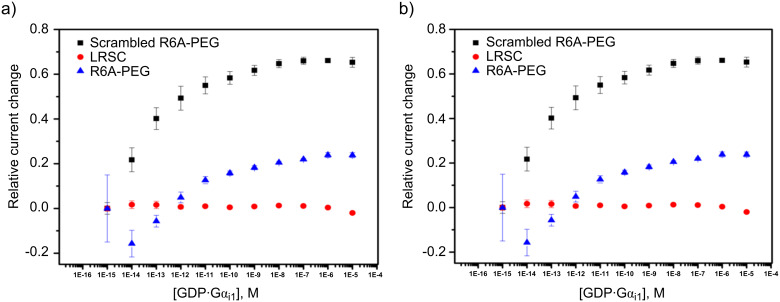
A failure in connection with the monitoring of binding events between a peptide and its G-protein target. a) Binding curve generated for Gα_i1_ binding to scrambled R6A peptide (black), LRSC peptide (red), and R6A peptide (blue). b) Binding curve generated after subtraction of nonspecific binding for Gα_i1_ binding to scrambled R6A peptide (black) and R6A peptide (red).

**Scheme 2:**
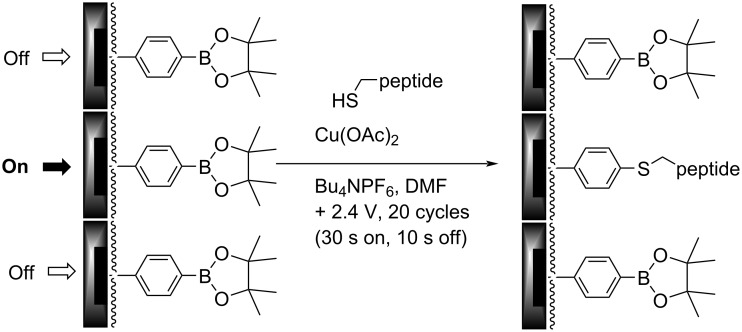
An array-based Chan–Lam coupling reaction.

However, the amplification of signals on the array could not be ignored as soon as we sought to use the arrays to guide the synthesis of YM- and FR-analogs as proposed in connection with Figure 1. YM and FR bind the alpha-subunit of the G-protein. So, we initially sought to show that the signaling approach being taken could monitor this type of interaction. The initial test was conducted with an R6A-peptide and its Gα_i1_-target (Figure 5) [21]. Gα_i1_ was selected as a positive control for subsequent studies because it can be expressed easily, has roughly 55% homology with the much more difficult to express Gα_q_ [15], and chimeric proteins are available that have Gα_i1_ modified with a Gα_q_ binding site for YM and FR [22]. R6A was chosen as the ligand for Gα_i1_ because it was known to bind the target with a binding constant of *K*_D_ = 60 nM [21]. The binding data shown in Figure 5 compared interactions between Gα_i1_ and two surface bound peptides; R6A (MSQTKRLDDQLYWWEYLC) and a scrambled R6A sequence (QLSEDTYLLMRWDYWQK). The two peptides were placed on the array along with a fluorescent dye (LRSC, lissamine rhodamine) that was used to make sure the placement chemistry was working. The array used was coated with the borate ester diblock copolymer (Figure 2), and the peptides were attached to this polymer through a PEG-6 linker in direct analogy to the RGD-peptide experiment shown above. In this case, a Cu(II)-mediated Chan–Lam coupling reaction was used to place each molecule on a arylborate ester coating the array (Scheme 2) [23]. The Cu(II) needed for the transformation was generated at the selected electrodes by the oxidation of a Cu(I) precursor, and then confined to the surface of those electrodes with the use of a solution-phase Cu(II)-mediated disulfide bond-forming reaction using excess substrate in solution. The molecules were placed by individually addressable blocks of electrodes on the array that were each comprised of 65 individual electrodes. This was done to increase the amount of current measured for the block of electrodes relative to many experiments that use blocks of 12 electrodes.

Once the molecules were in place, the array was placed in a buffer solution (see Supporting Information File 1 for details) containing ferrocene carboxylic acid as a redox mediator. Initially, the array was placed in a solution containing the smallest concentration of Gα_i1_ and then a current measured for the mediator at the electrodes in the array. The solution was then removed, replaced with a mediator/buffer solution containing the next highest concentration of Gα_i1_, and the current for the mediator again measured. This procedure was repeated for each concentration of Gα_i1_. From the data, it was clear that there was little binding to the electrodes proximal to the LRSC dye. At those electrodes, no change in current was evident as the concentration of receptor was varied. However, significant binding occurred for both the scrambled R6A and R6A itself. For the specific plots shown, we had been looking for the binding event to decrease the current. It did not. Instead, the binding event swelled the polymer, made it easier for the redox couple to reach the electrode, and thus caused an increase in the current. This led to an increase in current being shown as negative because it was opposite of what was planned and what we had seen in previous studies.

**Figure 6:**
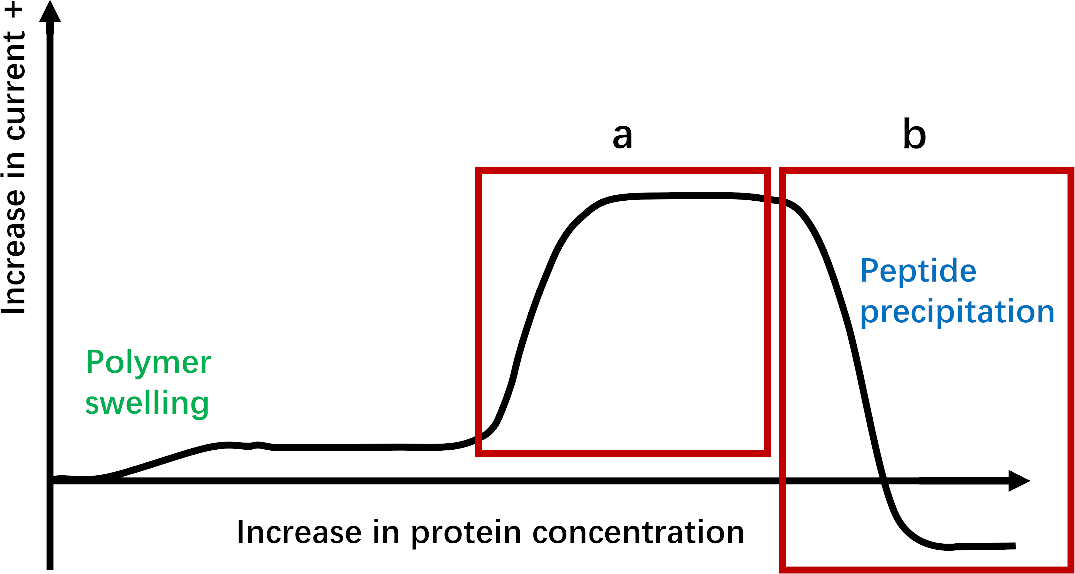
Potential for an immediate, rapid change in current. a) The binding event being monitored has already started. b) Precipitation of the protein target.

At this point, the non-specific binding of Gα_i1_ to the functionalized surface was removed from the data by subtracting the curve obtained for the scrambled peptide from both the scrambled peptide and R6A (Figure 5b). This revealed a clear, immediate change in current for the binding of Gα_i1_ to R6A. A rapid change in current of this nature can indicate either a binding event that is already occurring at the start of the experiment or a precipitation event (Figure 6). However, a precipitation event would cause a drop in current since precipitation of the protein onto a polymer-coated electrode prevents the mediator from reaching the electrodes below the polymer. Hence, the increase in current seen in the experiment shown in Figure 5b was associated with a binding event that was already occurring at the start of the experiment. The net result was that a full binding curve for the positive control selected for subsequent comparisons could not be observed. Hence, any subsequent evaluation of binding events using synthesized analogs of YM and FR could not quantified. A method for calibrating the positive control had to be found.

With the signal for the R6A/Gα_i1_ interaction amplified out of the window for the experiment, the question became what caused the amplification and how the problem could be remedied. Amplifications of surface-based signals are typically caused by the presence of multiple ligands for the receptor above any given electrode. If the protein dissociates from one ligand and then rapidly binds another on the surface of the electrode, then it never leaves the surface. The surface does not recover its original conductivity and the experiment shows a binding constant that is greater than either of the individual interactions. Alternatively, the surface-bound substrate could lead to avidity events where more than one of the ligands binds to the protein at the same time by taking advantage of allosteric binding sites.

Either way, the result is the same. The data shown in Figure 5 cannot be used to assess the binding strength of the interaction. The experiment needed to be calibrated so that the signal could be moved into the window where the full binding curve could be observed.

Both of the possible causes for amplification of the signal result from too high of a concentration of ligand on the surface of the electrodes. Solving the problem requires a reduction in the amount of peptide on the surface of the electrodes in the array. This can be accomplished in two ways. First, the amount of material placed on the array can be decreased by cutting the reaction time used for the placement reaction. The amount of material placed onto the surface of an array is linearly dependent on the time used for the placement reaction up until the surface becomes saturated [23]. Second, the peptide to be placed on the surface of the array can be diluted with a substrate that does not bind the receptor before placement on the array. For any given reaction time, this would lower the density of the active peptide ligand on the surface of the electrode where it was placed. Of course, a combination of the two methods can be used.

**Figure 7:**
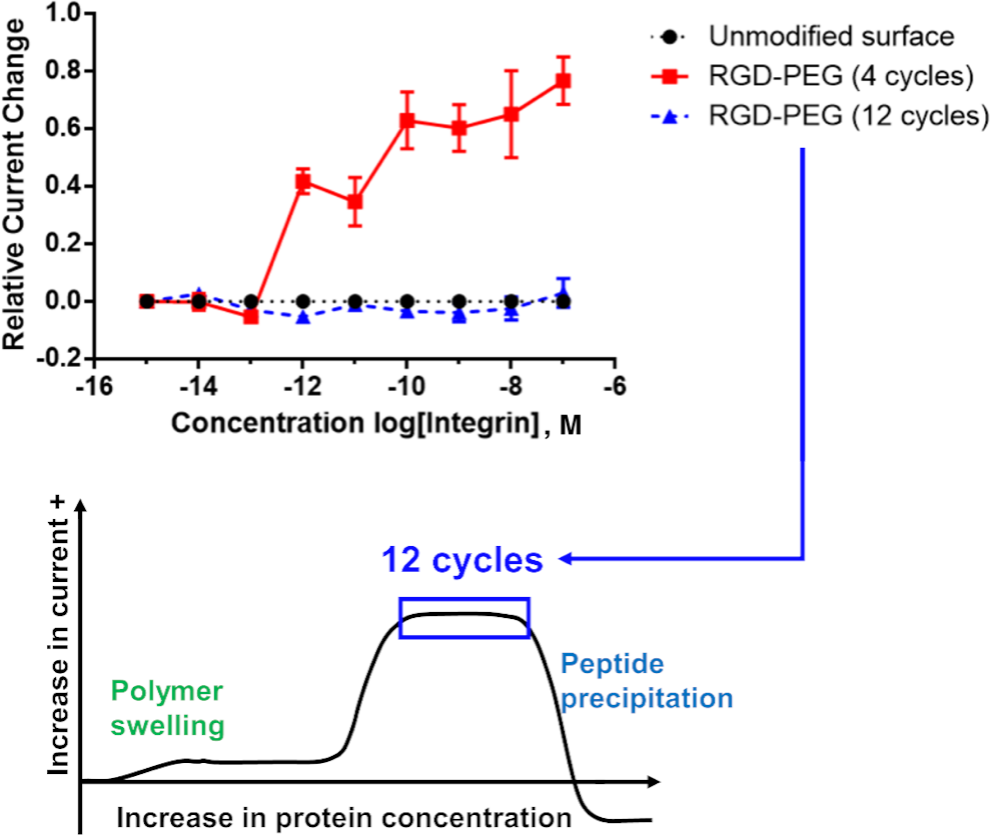
Four cycles vs. twelve cycles and the effect on binding curve location.

In order to probe the effectiveness of these two methods with a cheaper option than Gα_i1_, the effect of peptide surface concentration on the location of the binding curve obtained was initially studied with the more readily available RGD-peptide/integrin pair. Initially, the amount of RGD-peptide placed on the array was increased to see if we could mimic the experiment with the R6A/ Gα_i1_ pair that amplified the binding curve out of the observable window. This effort is highlighted in Figure 7.

For this experiment, the number of cycles used for placement of the RGD-PEG_6_ substrate onto the array was varied from 4 cycles (the initial experiment shown in Figure 4) to 12 cycles. As in the earlier experiments, each cycle involved turning the electrode on for 90 s and then off for 180 s. The more cycles used the longer the time employed for the placement reaction. The "binding curves" generated at the electrodes with these two placement times were compared with the background signal derived from the unfunctionalized polymer. The more stable (relative to iron-based mediator pairs) hydroquinone/quinone redox pair was used, again in direct analogy to the experiment shown in Figure 4. The current associated with this redox couple at each of the electrode in the array was again monitored by using the array as an anode and the remote Pt electrode in the electrolysis cell as a cathode. The surface of the array was the arylbromide-based diblock copolymer. Binding between the integrin receptor and the RGD peptide on the polymer increased the solubility of the polymer above the associated electrodes and in so doing increased the current associated with the mediator. This change in current relative to the concentration of integrin in solution is what is recorded in Figure 7. From here on in, all changes in current on the array will be plotted as positive since the increase in current was observed for every experiment conducted in this study.

The background signal measured using the unfunctionalized electrodes showed no signal. This indicated the absence of any significant non-specific binding event between the integrin receptor and the polymer coating on the array. When 4 cycles were used to place the RGD-peptide (with the C-PEG_6_ linker) on the surface of the electrodes, the subsequent analytical experiment led to a clear binding curve. Once again, the binding curve measured in this manner was amplified relative to the known nanomolar binding constant for the interaction. When three times the reaction time was used for the placement reaction (12 cycles), the binding curve was amplified out of the window. In this case, no signal could be observed. This was not a surprise since the experiment measures a change in current from the initial starting point. If the binding event is complete at the initial concentration, then no current change will be measured, and the experiment gives rise to an "curve" that cannot be distinguished from background.

**Figure 8:**
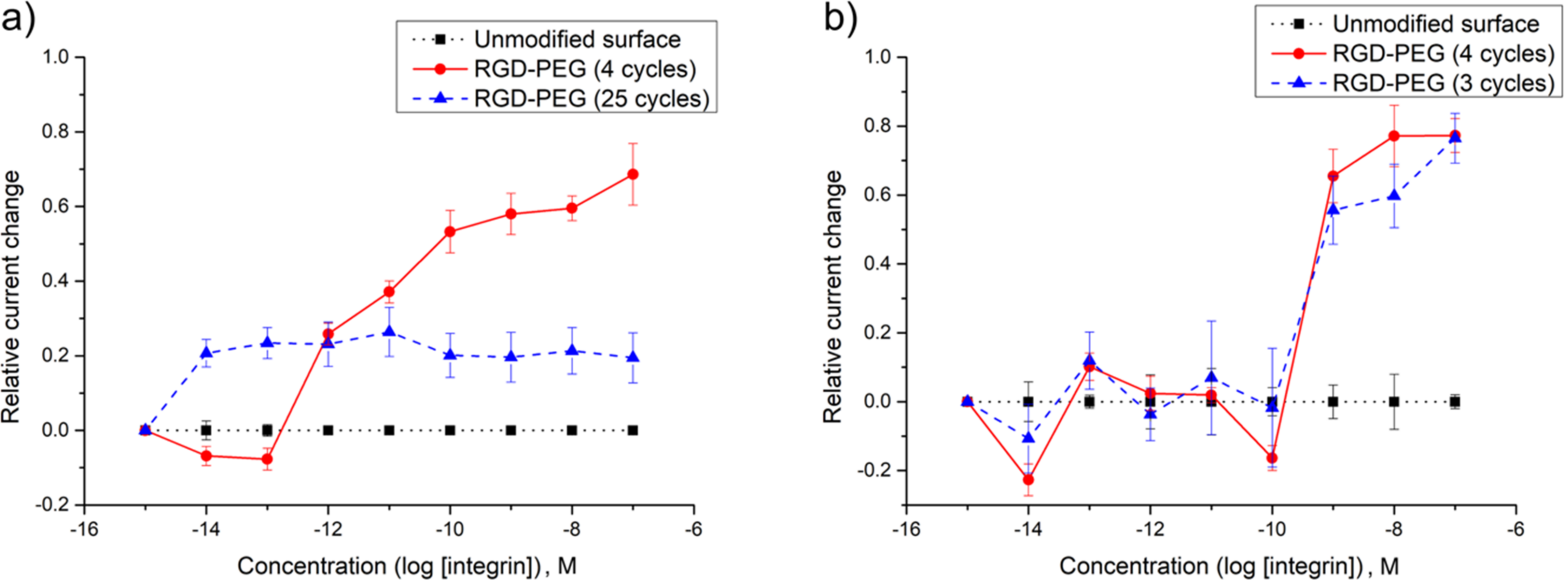
Repeating the experiment on different arrays. (a) A comparison between a 4-cycle placement reaction and a 25-cycle placement reaction. (b) 3 vs. 4 cycles.

The observation highlights why it is essential that all array-based studies contain a positive control that can be used to demonstrate that a binding event can be measured using the synthetic conditions employed. This is particularly important since the amount of material on the surface of an electrode is dependent not only on the length of time for the placement experiment, but also on the surface itself. A thicker polymer coating affords more attachment sites for the peptide placed on the array, a thinner polymer fewer. Hence, the surface coverage for a given placement reaction is dependent both on the time used for the experiment and the thickness of the polymer coating. While the thickness of the surface was initially characterized for the coating of an array [17], that thickness is not perfectly reproducible from one array to the next. Consider the two different signaling experiments shown in Figure 8. Both were performed in a manner identical to the experiment shown in Figure 7. In the first experiment, a single array was used to compare a placement reaction conducted for 4 cycles with one conducted for 25 cycles. The two experiments were conducted at different regions of the array. The binding curve for the experiment using 4 cycles closely resembled the experiment shown in Figure 7. However, there was a significant difference with the experiment using a longer placement reaction. In this case, part of the binding curve could be seen. The lower total change in current observed was due to the fact that only the top piece of the binding curve was observed with much of the binding curve remaining out of the window of the experiment. Hence the total change in current from the starting point of the experiment was smaller. When compared to the earlier experiment shown in Figure 7, the binding curve was still shifted out of the window of the experiment with the longer placement reaction but not as far as the earlier experiment. The difference was due to the change in the coating on the array. A thinner polymer coating on the portion of the array used for the 25-cycle placement reaction would mean less material on the surface of the array, even relative to a different array using 12 cycles for the placement reaction. So, the overall conclusion of the two experiments was the same. The location of the binding curve could be calibrated so that the entire binding curve could be seen by controlling the amount of peptide on the surface of the electrodes. But, the specific details of the experiments were not identical.

The same conclusion was reached with the second experiment shown in Figure 8. In this case, the difference between the placement reactions was kept small in order to see how sensitive the experiment was to the placement reaction. The electrodes functionalized with a placement reaction using 4 cycles and the electrodes functionalized with a placement reaction using 3 cycles gave rise to almost identical binding curves that could be seen in their entirety. However, those binding curves were not in the same location as that observed for the experiment in Figure 7. The polymer thickness on this array was again thinner leading to peptide on the surface of the electrodes.

Clearly, the method is not compatible with providing an absolute measure for a binding constant in the absence of a positive control. There is simply too much variance in the coating of the surface from one array to the next. Fortunately, it was also clear from the observations that once an experiment was calibrated to place a positive control in the center of observable window, variations in the surface from experiment to experiment will not move the positive control out of that window.

**Figure 9:**
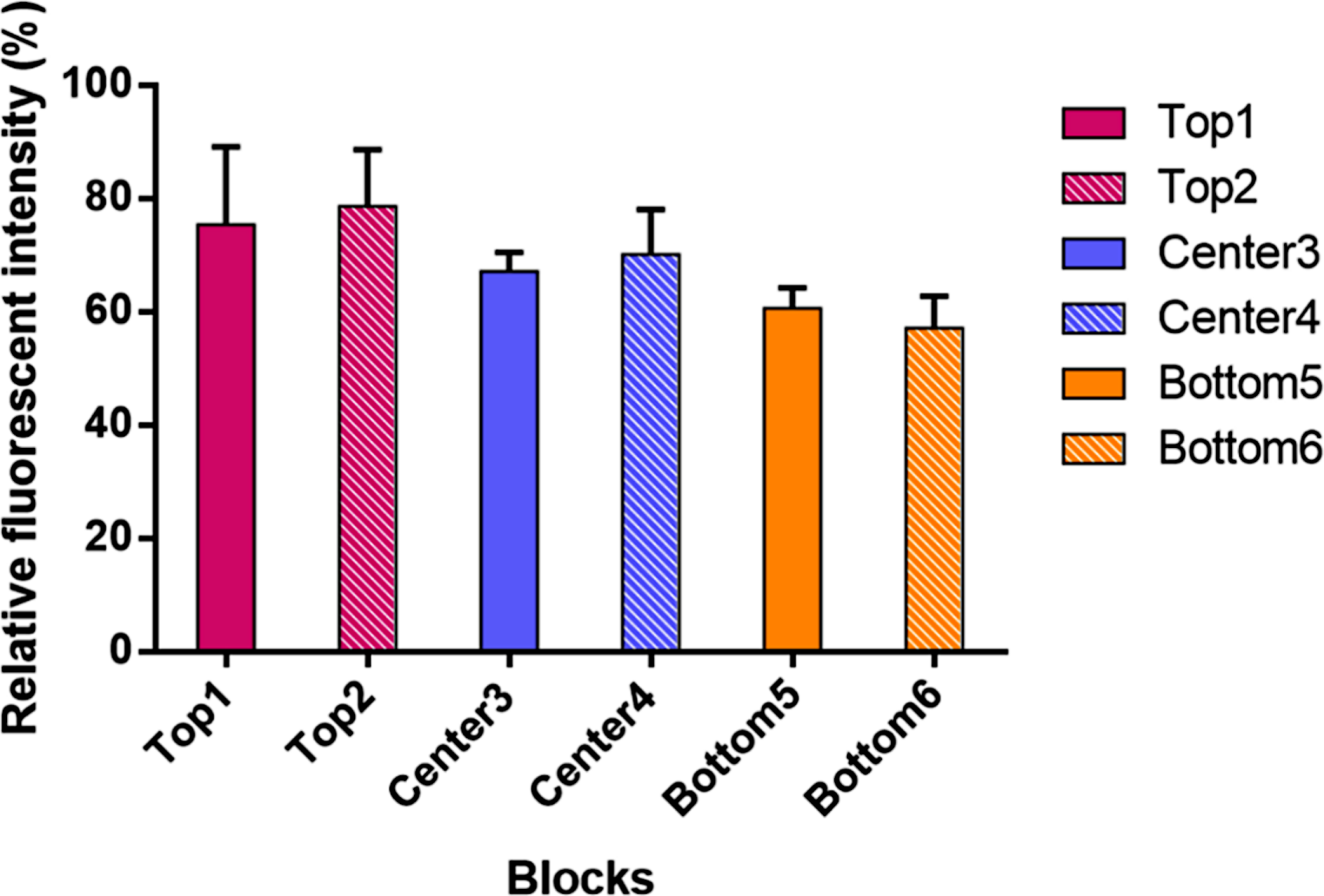
Quantitative fluorescent study on variance of the polymer coating across the microelectrode surface. Purple represents blocks from the top region. Blue represents blocks from the center region. Orange represents blocks from the bottom region.

Evidence for the variations on a single array being relatively small is shown in Figure 9. In this experiment, a 12K array was coated with the arylbromide-based diblock copolymer and then blocks of 12 electrodes each functionalized with pyrene-butanol at various places on the array. For the placement reaction, the Cu(I)-catalyzed cross-coupling reaction used above (Scheme 1) was employed for 4 cycles (90 s on and 180 s off). The chemistry placed enough of the alcohol by the electrodes for the pyrene to form the exciplex dimer that fluoresces in the red region of the spectrum and does not self-quench. A fluorescence microscope was then used to take an image of the array, and the image used to quantify the fluorescence, and thereby the amount of material present, at each region of the array. The process was repeated three times on the same array in order generate an average intensity for each region examined. Two blocks of electrodes were examined from the top, center, and bottom regions of the array. While the data did not vary to a large extent, there were differences in the amount of material placed at the top, center, and bottom regions of the array. Variations within the regions were smaller. Since all of the placement reactions were run at the same time for the same duration, the only difference between the sites examined was a lack of uniformity in the polymer coating on the array. From a practical standpoint, the variance in the nature of the surface on an array translates into signaling studies with smaller error bars if a single region of the array is used for any given analysis. With a total of 12,544 electrodes on the array, this is not typically a problem. There are more than enough electrodes in any one region of the array for an extensive study.

With that information in place, attention was turned back to the initial small molecule/G-protein interaction that lies at the heart of guiding the synthetic study proposed in connection with Figure 1. Initially, the same approach used for the RGD/integrin model system was employed for this study. Namely, the amount of the R6A-peptide placed on the array was controlled by the number of cycles used for the placement reaction. The R6A-peptide was first attached to a C-PEG_6_-linker, and then the Cu(I)-catalyzed reaction used for its placement on an array coated with the arylbromide-based diblock copolymer. The reaction conditions for the placement reaction were identical to those described above.

**Scheme 3:**
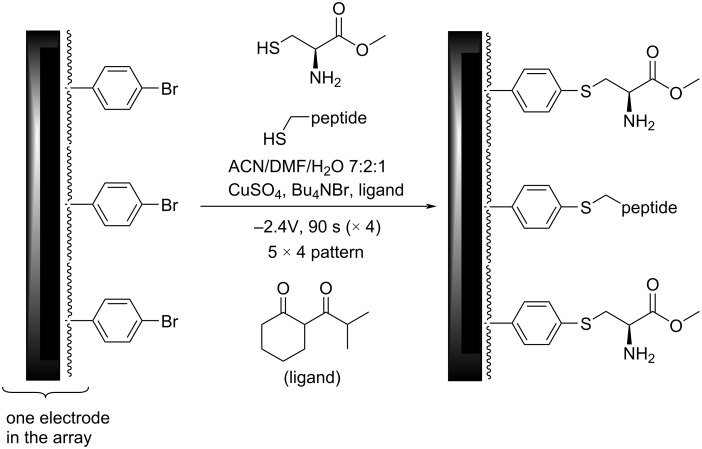
A new method for decreasing the concentration of R6A on the surface of the electrodes.

In this case, the effort to shift the curve into the window of the experiment was not successful. No matter how few cycles were used for the placement reaction, the full binding curve for the interaction between R6A and its Gα_i1_-target could not be observed. A method was needed to further reduce the concentration of the R6A peptide on the surface of the electrodes. To this end, cysteine methyl ester was selected as a molecule with which to dilute the R6A peptide because cysteine methyl ester does not show any background binding to Gα_i1_, and it can be efficiently placed onto the arylbromide-based surface using the same Cu(I)-catalyzed cross-coupling reaction used to place the R6A-PEG-C substrate on the array (Scheme 3).

**Figure 10:**
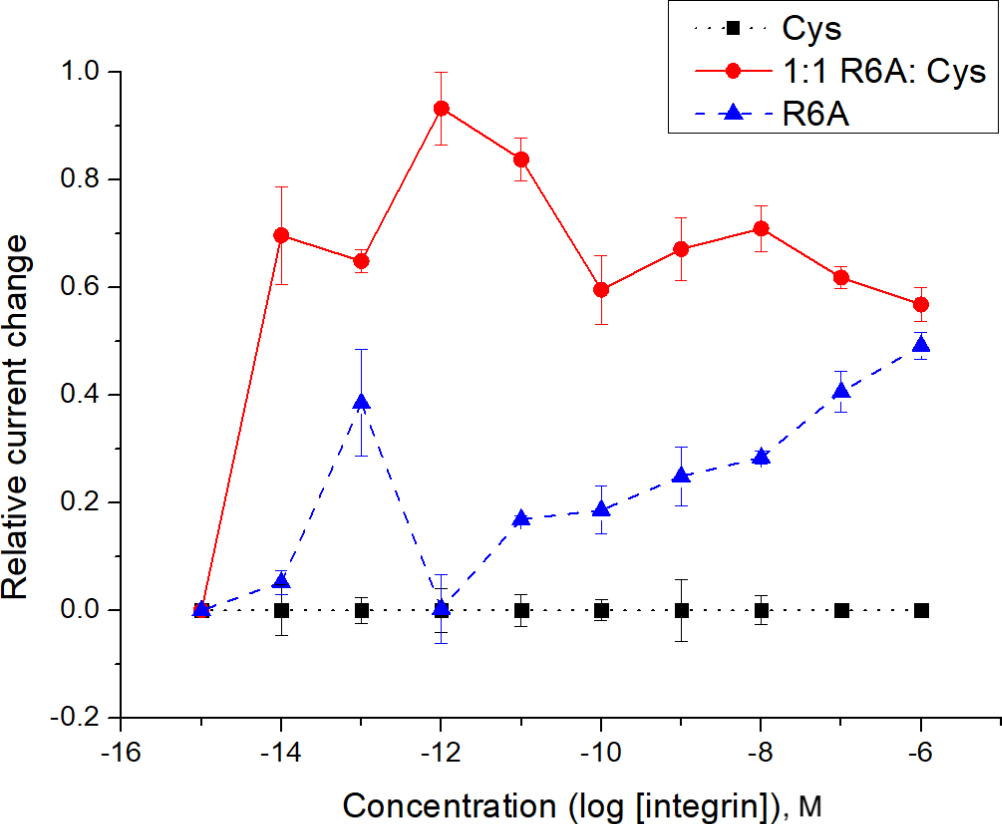
An initial study and the comparison of an R6A surface and a 1:1 R6A/cysteine methyl ester surface.

The first attempt at the dilution study compared a set of control electrodes that had only the cysteine methyl ester placed on the polymer coating their surfaces, a set of electrodes that were functionalized with only the R6A-PEG-C substrate, and a set of electrodes that were functionalized with a 1:1 mixture of R6A-PEG-C to cysteine methyl ester (Figure 10). The placement reactions were conducted for 4 cycles using the method described above.

The analytical study used the hydroquinone/quinone redox couple as the mediator and then added increasing amounts of the Gα_i1_-protein in solution as indicated in Figure 10. The electrodes functionalized with only cysteine showed no interaction with Gα_i1_. Those electrodes were used as a baseline to subtract any non-specific binding from the other curves. The signal from the electrodes functionalized with only the R6A-PEG substrate showed negligible binding over the background indicating that the signal was amplified completely out of the window for the experiment. A significant binding event was observed at the electrodes functionalized with a 1:1 mixture of R6A-PEG and cysteine, although it was still shifted out of the window so that only the latter portion of the binding curve could be observed.

**Figure 11:**
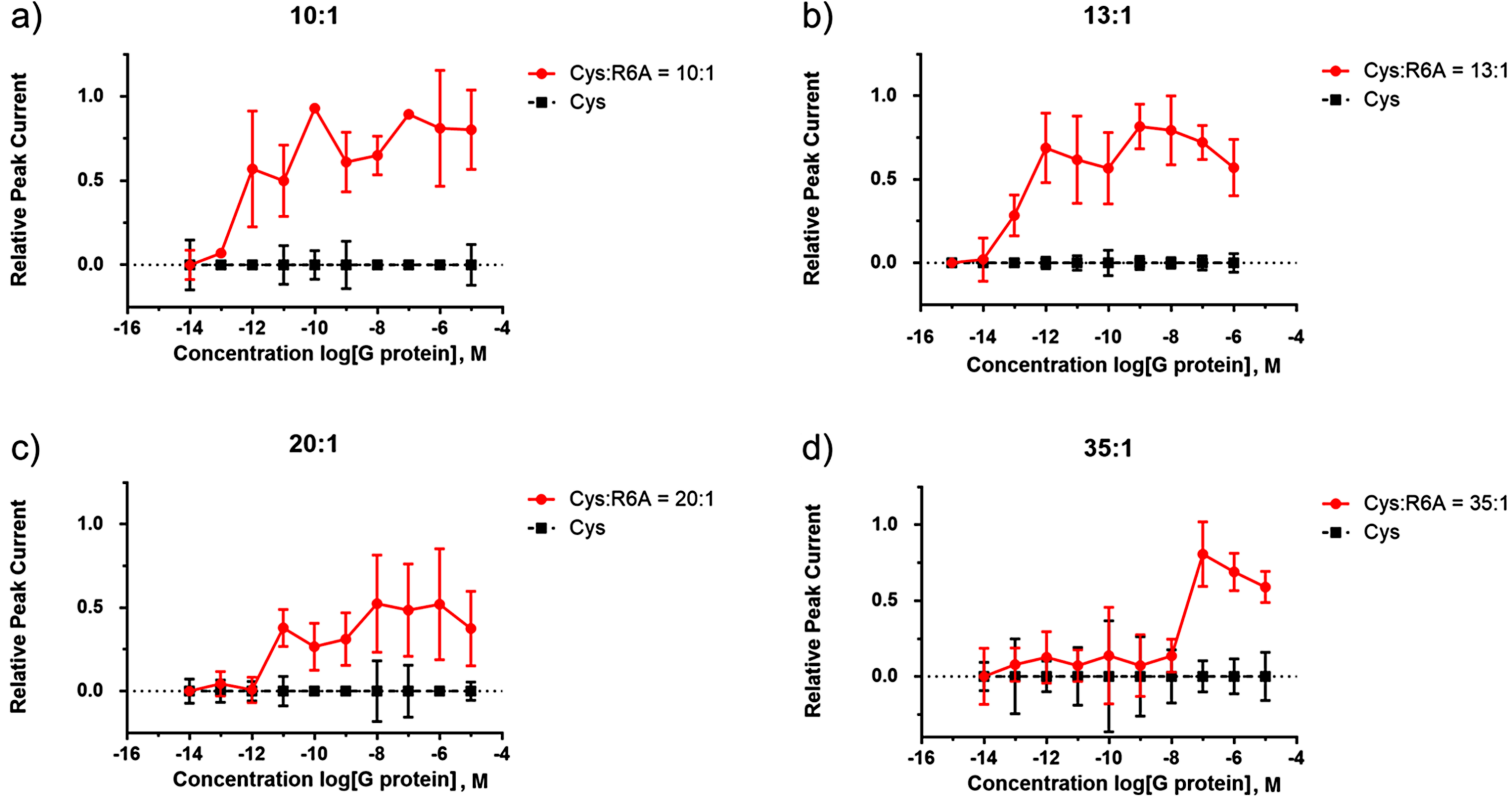
Calibrating the array-based signaling experiment for monitoring small molecule G-protein interactions.

With this initial experiment in place, the ratio of cysteine to the R6A-PEG substrate was varied (Figure 11). The experiments shown in Figure 11a and b were run on different arrays by different individuals at different times. The two experiments used very similar dilutions, and they afforded very similar results. The full binding curve could be seen for each experiment although both were close the edge of the window. It did appear that for the R6A-Gα_i1_ small variations in the surface of the array were not a significant issue. Further dilution of the R6A peptide with cysteine methyl ester (Figure 11c and d) then illustrated how the location of the binding curve could be moved within the window of the experiment, an observation that shows how the array experiment can be calibrated in order to place the positive control where it can best be used for comparison with other interactions involving Gα_i1_. Further adjustments could be made to place the signal in the middle of the window, but that level of optimization was deemed unnecessary for a model study.

## Conclusion

Indirect measurements on microelectrode arrays have two consistent issues. One is that the signals are amplified by either avidity effects or multiple binding events happening on the surface of an electrode. The second is that variations between arrays and the coatings placed on the arrays can cause shifts in the binding constants measured for any given interaction. These two problems can both be addressed by controlling the concentration of a ligand on the surface of the electrodes, something that can be readily accomplished using an electrosynthetic reaction. This allows for calibration of a signaling experiment and the placement of a positive control in the center of the observable window. The result is an opportunity to measure relative binding constants for ligands for a given receptor. The results presented set the stage for using microelectrode arrays to guide synthetic efforts aimed at probing G-protein/peptide interactions.

## Supporting Information

File 1 Procedures for electrolysis and cyclic voltammetry experiments, characterization of electrolysis products, procedures for synthesis and characterization of electrolysis starting materials.

## References

[1] Chandra S, Siraj S, Wong D K Y (2017). ChemElectroChem.

[2] Lee E-j, Chan E W L, Luo W, Yousaf M N (2014). RSC Adv.

[3] Li J, Sun C-L, An P, Liu X, Dong R, Sun J, Zhang X, Xie Y, Qin C, Zheng W (2019). J Am Chem Soc.

[4] Soscia D A, Lam D, Tooker A C, Enright H A, Triplett M, Karande P, Peters S K G, Sales A P, Wheeler E K, Fischer N O (2020). Lab Chip.

[5] Stuart M, Maurer K, Moeller K D (2008). Bioconjugate Chem.

[6] Fellet M S, Bartels J L, Bi B, Moeller K D (2012). J Am Chem Soc.

[7] Graaf M D, Marquez B V, Yeh N-H, Lapi S E, Moeller K D (2016). ACS Chem Biol.

[8] Dill K, Montgomery D D, Wang W, Tsai J C (2001). Anal Chim Acta.

[9] Bartels J, Lu P, Maurer K, Walker A V, Moeller K D (2011). Langmuir.

[10] Graaf M D, Moeller K D (2015). Langmuir.

[11] Yeh N-H, Zhu Y, Moeller K D (2019). ChemElectroChem.

[12] Sheridan M V, Lam K, Sharafi M, Schneebeli S T, Geiger W E (2016). Langmuir.

[13] Karbelkar A A, Furst A L (2020). ACS Infect Dis.

[14] Wehmeyer K R, White R J, Kissinger P T, Heineman W R (2021). Annu Rev Anal Chem.

[15] Nishimura A, Kitano K, Takasaki J, Taniguchi M, Mizuno N, Tago K, Hakoshima T, Itoh H (2010). Proc Natl Acad Sci U S A.

[16] Tietze D, Kaufmann D, Tietze A A, Voll A, Reher R, König G, Hausch F (2019). J Chem Inf Model.

[17] Hu L, Bartels J L, Bartels J W, Maurer K, Moeller K D (2009). J Am Chem Soc.

[18] Hu L, Graaf M D, Moeller K D (2013). J Electrochem Soc.

[19] Yeh N-H, Medcalf M, Moeller K D (2018). J Am Chem Soc.

[20] Gulaboski R, Bogeski I, Mirčeski V, Saul S, Pasieka B, Haeri H H, Stefova M, Stanoeva J P, Mitrev S, Hoth M (2013). Sci Rep.

[21] Ja W W, Roberts R W (2004). Biochemistry.

[22] Onken M D, Makepeace C M, Kaltenbronn K M, Kanai S M, Todd T D, Wang S, Broekelmann T J, Rao P K, Cooper J A, Blumer K J (2018). Sci Signaling.

[23] Graaf M D, Moeller K D (2016). J Org Chem.

