# Supplementary material for: Microelectrode arrays, electrosynthesis, and the optimization of signaling on an inert, stable surface
Source: Beilstein J Org Chem. 2022 Oct 20;18:1488–98. doi: 10.3762/bjoc.18.156 (PMC9592966; doi:10.3762/bjoc.18.156)
Supplement: File 1 — Procedures for electrolysis and cyclic voltammetry experiments, characterization of electrolysis products, procedures for synthesis and characterization of electrolysis starting materials. [file Beilstein_J_Org_Chem-18-1488-s001.docx]

**Microelectrode Arrays, Electrosynthesis, and the Optimization of Signaling on as Inert, Stable Surface.**

Kendra Drayton-White,^&^ Siyue Liu,^&^ Yu-Chia Chang, Sakshi Uppal, and Kevin D. Moeller*

Washington University in Saint Louis, Saint Louis, Missouri 63130, United States

Email: moeller@wustl.edu

* Corresponding author

^&^ The two authors contributed equally to this work.

**Supporting information**

**General:**

**Reagents**

Cysteine methyl ester hydrochloride was purchased from Chem-Impex International, Inc. (Wood Dale, IL). Copper(II) Sulfate, 2-isobutrylcyclohexanone, tetrabutylammonium bromide, acetonitrile, N,N-dimethylformamide and phosphate buffered saline (PBS) solution tablet were purchased from Sigma-Aldrich (St. Louis, MO). G-protein with a concentration of 3.569 ng/nL was purified from *E.coli* using the published procedures [1].

Biological reactants: GRGDSP peptide was purchased from Sigma-Aldrich (St. Louis, MO). Custom linker peptide (C-PEG6-GGRGDGP) and R6A customized peptide (Ac-MSQTKRLDDQLYWWEYL-PEG6-C-NH2) were chemically synthesized by CPC Scientific, Inc. (San Jose, CA) and purified by HPLC. Alpha 5, beta 1-integrin was purchased from AcroBiosystems (Newark, DE).

**Materials**

Microelectrode arrays and the ElectraSense reader / power supply were provided by CustomArray, Inc. (Bothell, WA). The reader is used to activate the selected electrodes on the array for cyclic voltammetry measurements, and an external BAS 100B Electrochemical Analyzer controls the potential sweep.

For the quantitative fluorescent study on the polymer coating, a Nikon Eclipse E200 microscope, a Boyce Scientific M-100 burner, and a Nikon D5000 camera were used for fluorescence microscopy. The following optical filters were used: ET-GFP (FITC/Cy2) (Chroma), CFW-BP01-Clinical-000 (Semrock) filter cube excitation 380-395 nm/emission 420-470 nm, and TxRed-A-Basic-000 (Semrock) filter cube excitation 540-580 nm/emission 590-670 nm.

**Sample procedure for spin-coating arrays with diblock copolymer**

The microelectrode arrays were coated with a diblock copolymer using a model WS-400B-6NPP/ LITE spin-coater. The array chip was inserted into the spin-socket cap's and taped to the cap to secure it, covering the pronged portion of the array but leaving the electrodes uncovered. The spin-cap was then inserted into the spinner and positioned horizontally. To completely cover the electrode area with diblock copolymer on the chip, four drops of a 0.03 g/mL poly(4-bromostyrene)-block-poly (2-cinnamoylethyl methacrylate) (PBrSt-b-PCEMA) solution in a xylene/THF solution ratio were added. The chip was then spun at 1000 rpm for 40 s under nitrogen. Prior to usage, the coated array chip was irradiated with a 100W Hg lamp for 20 minutes after drying for three minutes under nitrogen.

Characterization of diblock copolymer:

^1^H Spectrum for diblcok copolymer

According to NMR spectrum,

Block A: DPn= {23.00-26.09/4)/2}/ (1/6) = 49

Block B: DPn= (26.09/4)/ (1/6) = 39

Molecular weight of diblock copolymer: 19271.23 g/mol

**Preparation of reaction solution for synthetic experiment on the array by Cu(I)-catalyzed coupling reactions**

Preparation of single peptide solution

Peptide (RGD-PEG/R6A-PEG/ 5.0-8.0 mg) and 5.0-8.0 mg of tetrabutylammonium bromide were dissolved into 100 µL of DMF in an Eppendorf tube. Seven µL of a 25 mM copper(II) sulfate solution in DI water and 7µL of 50 mM 2-isobutrylcyclohexanone solution in DMF were added to the same tube. This mixture was added to another tube with 1.5 mL of a 7:2:1 solution of MeCN/DMF/water.

Preparation of diluted peptide reaction solution with cysteine methyl ester

A total 8 mg of peptide(RGD-PEG/R6A-PEG) and cysteine methyl ester mixture with a certain mass ratio, and 5.0-8.0 mg tetrabutylammonium bromide were dissolved into 100 µL of DMF in an Eppendorf tube. Seven µL of 25 mM copper(II) sulfate solution in DI water and 7µL of 50 mM 2-isobutrylcyclohexanone solution in DMF were added to the same tube. This mixture was added to another tube with 1.5 mL mixture solution of 7:2:1 MeCN/DMF/water.

Preparation of cysteine methyl ester solution

Cysteine methyl ester (5.0-8.0 mg) and 5.0-8.0 mg tetrabutylammonium bromide were dissolved into 100 µL of DMF in an Eppendorf tube. Seven µL of 25 mM copper(II) sulfate solution in DI water and 7µL of 50 mM 2-isobutrylcyclohexanone solution in DMF were added to the same tube. This mixture was added to another tube with 1.5 mL mixture solution of 7:2:1 MeCN/DMF/water.

**Synthetic experiment on the array by Cu(I)-catalyzed coupling reactions** (Since we have found it preferable to use the Cu(I)-catalyzed cross coupling with arylbromide surfaces preferrable to the Chan-Lam procedure, only the Cu(I)-base procedure is reported here. For a detailed description of the Chan-Lam procedure please see the references cited in the text).

A 110-120 µL reaction solution was added to the array. Ten blocks of 12 electrodes (4X3 pattern) were selected and set to a potential of -2.4V relative to the auxiliary electrode for a certain number of reaction cycles (1 cycle refers to 90s on and 180 s off). The array was washed extensively with 95% ethanol after completion of the reaction.

**Preparation and storage of 10^-15 M to 10^-6 M Intergin mediator solution**

Intergin (100 µg) was dissolved in 1 mL of an 8 mM quinone redox mediator solution to obtain 0.514x10^-6 M to 10^-15 M protein solution. Series of dilutions were made sequentially. Liquid nitrogen was filled in each eppendorf tube with ten gradient concentrations of protein solution which were transferred to -80℃ freezer immediately for storage.

**Preparation and storage of 10^-15 M to 10^-6 M Gi1 mediator solution**

Gi1 protein (71.4 μg) was dissolved in 0.165 mL of an 8 mM quinone redox mediator solution at a final concentration of 0.01 mM. Series of dilutions were made sequentially from 10^-6 M to 10^-15 M. Ten different protein solution concentrations in each eppendorf tube were put into liquid nitrogen before being immediately frozen at -80°C for storage.

**Preparation of 8mM quinone redox mediator buffer solution for both protein solution and polymer saturation**

Benzoquinone (17.3 mg) and 17.6 mg of hydroquinone were dissolved in 20 mL PBS buffer solution. The mixture was sonicated until completely dissolved. The redox mediator solution was made freshly for each experiment and protein solution.

**CV Signaling experiment procedure:**

When the array coated with the peptide functionalized polymer was ready, a series of protein solutions with gradient concentrations were taken out from the freezer, thawed by hand immediately, and stored at 0℃ during the whole time of the experiment. The electroanalysis started by running cyclic voltammetry at all of the electrodes in the array (a whole board pattern) using a quinone buffer solution for saturation of the polymer. The potential at electrodes was scanned from -700 mV to +700 mV relative to the Pt-cap over the array at a scan rate of 400 mV/s. For the subsequent analytical experiments, the same procedure was conducted at selected electrodes. The current at the electrodes was measured and recorded as a function of the applied voltage. For each experiment, the measurement was repeated for 2-3 times until the peak current stayed stable. The experiment looked at three to six blocks of electrodes functionalized with peptide (and co-substrate). Following data collection for each concentration of a receptor, the array was washed with 95% ethanol and dried and the measurement was repeated with the next concentration of protein. The procedure was repeated until a CV had been recorded at each of the selected blocks for each concentration of protein.

**Data processing procedure**

The peak current passing through the selected electrodes was measured using the difference between the peaks of the oxidative wave and the reductive wave. The peak current was measured and calculated for each selected electrode. The values in the dataset were normalized to be between zero to one, with the largest peak current data point have a normalized value of one. The error bars came from the differences among three sets of selected electrodes. The data were normalized respectively for three of them. After that, average and standard errors were calculated and plotted as a function of the logarithm of the protein concentration (M). The data was processed by GraphPad Prism 6 and OriginPro 9 64-bit.

**Procedure for polymer surface variation by fluorescence.**

The aryl bromide based diblock copolymer was coated on a 12K array. Pyrene-butanol was coated on the whole board of the array with 4 cycles of Cu(I) catalyzed reaction. Fluorescence pattern pictures of 3 different areas (top, center, bottom) , Six different blocks on the array were examined (top1, top2, center3, center4, bottom5, bottom6) with a fluorescence microscope. For each block, fluorescence of 12 electrodes (4 x 3 pattern) was measured by ImageJ. The experiment was repeated 3 times on the same array. Each time, for the same area, the 12 selected electrodes of blocks were varied. Average data and standard error of relative fluorescent intensity were calculated.

**Reference**

[1] Onken, M. D.; Makepeace, C. M.; Kaltenbronn, K. M.; Kanai, S. M.; Todd, T. D.; Wang, S.; Broekelmann, T. J.; Rao, P. K.; Cooper, J. A.; Blumer, K. J. Targeting Nucleotide Exchange to Inhibit Constitutively Active G Protein α Subunits in Cancer Cells. *Science Signaling****.* 2018**, *11*, 546.
